# Evaluation of antioxidant status and lipid peroxidation in dogs with myxomatous mitral valve degeneration stage B1

**DOI:** 10.3389/fvets.2023.1203480

**Published:** 2023-09-06

**Authors:** Katerina Tomsič, Aleksandra Domanjko Petrič, Ana Nemec, Tatjana Pirman, Vida Rezar, Alenka Seliškar, Tomaž Vovk, Alenka Nemec Svete

**Affiliations:** ^1^Small Animal Clinic, Veterinary Faculty, University of Ljubljana, Ljubljana, Slovenia; ^2^Department of Animal Science, Biotechnical Faculty, University of Ljubljana, Domžale, Slovenia; ^3^The Chair of Biopharmaceutics and Pharmacokinetics, Faculty of Pharmacy, University of Ljubljana, Ljubljana, Slovenia

**Keywords:** myxomatous mitral valve degeneration, antioxidant status, lipid peroxidation, oxidative stress, dog

## Abstract

Myxomatous mitral valve degeneration (MMVD) is the most common naturally occurring heart disease in dogs. There is a lack of data on antioxidant status and oxidative damage in dogs with MMVD stage B1 according to the American College of Veterinary Internal Medicine (ACVIM B1). The aim of this study was to investigate antioxidant status (plasma vitamin E, lipid-standardized vitamin E (LS-VitE), antioxidant capacity of lipid-(ACL) and water-soluble antioxidants, whole blood glutathione peroxidase and erythrocyte superoxide dismutase), and lipid peroxidation [malondialdehyde (MDA)] in dogs with MMVD ACVIM B1. Serum cholesterol and triglyceride concentrations were measured to calculate LS-VitE. Fourteen dogs with MMVD ACVIM B1 and 12 control dogs were included in the study. Dogs with MMVD had significantly higher vitamin E, ACL, MDA, and cholesterol concentrations and significantly higher LS-VitE values than control dogs. No significant correlations between MDA and antioxidant parameters were determined in either group. In conclusion, oxidative damage to lipids is already present and the antioxidant status is altered but not depleted in dogs with MMVD ACVIM B1. The antioxidant response to increased oxidative damage consists mainly of the activation of fat-soluble antioxidants. Further research is needed to evaluate the efficacy and targets of early antioxidant supplementation to prevent or ameliorate oxidative stress and mitigate disease progression in dogs with early-stage MMVD.

## 1. Introduction

Myxomatous valve degeneration is a progressive degenerative disease of the heart valves that occurs most commonly in small to medium-sized older dogs and mainly affects the mitral valve (1–4). The disease is characterized by structural changes that result in thickening of the valve leaflets, compromising their complete apposition and leading to leakage and regurgitation. The resulting ventricular and atrial overload leads to remodeling of the heart and alteration of the myocardium (1, 2, 5).

Although myxomatous mitral valve degeneration (MMVD) is the most common cardiac disease in dogs, its etiopathogenesis is not yet fully understood. Polygenetic factors are thought to be responsible for the development of the disease due to the high incidence in small to medium-sized dogs and in some dog breeds, such as the Cavalier King Charles Spaniel (1–3). Some studies suggest the possible role of the serotonin signaling pathway, which is thought to be activated by mechanical and physical stress on the heart valves during cardiac work (2–5). Oxidative stress, defined as “an imbalance between oxidants and antioxidants in favor of oxidants, resulting in impaired disruption of redox signaling and control and/or molecular damage” (6), has also been linked to cardiac remodeling and myocardial damage in humans (7). Oxidative stress is also associated with the inflammatory response of cardiac cells, which has already been confirmed in dogs with heart failure (8, 9) and in dogs with different stages of MMVD (10).

Cardiac tissue is constantly exposed to the formation of reactive oxygen species (ROS), which are highly reactive products of aerobic metabolism involved in cellular signaling, adaptation to stress (11, 12), and immune response (13). Due to their reactive nature, ROS can interact with and alter biologically important molecules such as lipids, proteins, and DNA (11). Malondialdehyde (MDA), for example, a low molecular weight aldehyde, is formed as a by-product of lipid peroxidation. It is commonly used as a marker of oxidative lipid damage and thus represents a marker of oxidative stress. Despite its enzymatic degradation, MDA can react and damage cell and tissue proteins or form DNA adducts with DNA bases (14, 15).

Under physiological conditions, ROS are in a dynamic equilibrium with antioxidant defenses that prevent or eliminate oxidative damage to molecules. Vitamin E is a scavenger of the free radicals that are produced in the chain reaction of lipid peroxidation, particularly in cell membranes (16). In addition to its antioxidant effect, vitamin E influences the cellular response to oxidative stress by altering signal transduction pathways (17) and is also implicated in the modulation of the inflammatory response of cardiac cells (18).

Superoxide dismutase (SOD) and glutathione peroxidase (GPX) are major endogenous antioxidant enzymes that counteract the pro-oxidant actions of ROS thereby protecting cells against the ROS-induced damage. Superoxide dismutase represents a family of metalloproteinase enzymes catalyzing the dismutation of superoxide into hydrogen peroxide (19). Glutathione peroxidases are a family of phylogenetically related enzymes catalyzing the reduction of hydrogen peroxide and other peroxides to oxygen and water, converting the co-substrate glutathione to an oxidized form (20).

The antioxidant capacity of plasma or other body fluids is a direct indicator of the influence of the environment, metabolism, physiological or pathological state, and dietary antioxidant intake on the redox status of the organism (21, 22). Water-soluble antioxidants (uric acid, ascorbic acid, albumins, and other low molecular weight antioxidants) are retained in the aqueous regions of the plasma, while lipid-soluble antioxidants (e.g., vitamin E, ubiquinone, and carotenoids) are hidden in lipoproteins (22).

Many studies have demonstrated the presence of oxidative stress in dogs with advanced MMVD and heart failure (8, 9, 23–28). Recent studies have investigated the influence of antioxidant supplementation on parameters of oxidative stress in dogs with MMVD (29–32). Nevertheless, few studies have examined changes in antioxidant status in dogs with early-stage MMVD (24, 25, 27, 29). Current therapeutic approaches target the effects of valvular dysfunction in dogs with clinically prominent symptoms of MMVD (2,  5). Therefore, the importance of early intervention can be supported by evidence of molecular changes that may exacerbate or worsen the disease.

We hypothesized that antioxidant status is altered and oxidative damage to lipids is already present in dogs with early-stage MMVD (ACVIM stage B1). This study aimed to investigate the levels of selected antioxidant parameters (antioxidant status), namely vitamin E, lipid-standardized vitamin E (LS-VitE), antioxidant capacity of lipid-(ACL) and water-soluble antioxidants (ACW), intracellular antioxidant enzymes GPX and SOD, and lipid peroxidation (assessed by measurement of MDA concentration) in a cohort of client-owned dogs with early-stage MMVD. The secondary aim was to investigate the relationship between the degree of lipid peroxidation (MDA) and individual parameters of antioxidant status.

## 2. Materials and methods

### 2.1. Animals

Dogs scheduled for periodontal treatment at the Small Animal Clinic (Faculty of Veterinary Medicine, Ljubljana, Slovenia) were enrolled in the study after informed consent from their owners was obtained. National Ethics Committee approved the research. All procedures were conducted in accordance with the Slovenian Animal Protection Act (Official Gazette of the Republic of Slovenia, 43/2007).

Dogs were fasted 12 h before blood collection, while water was available until arrival at the clinic. The health status of the dogs was assessed by history, clinical examination, as well as hematological and biochemical analysis (data not presented). A prerequisite for inclusion in the study was the absence of chronic systemic diseases (allergies, chronic nephropathies, endocrinopathies, hepatopathies, oncological diseases), acute diseases (infectious diseases, injuries, inflammation), and other cardiac diseases except MMVD ACVIM B1 (according to the classification of the American College of Veterinary Internal Medicine; ACVIM; MMVD group) (1) or the absence of heart disease (control group). Exclusion criteria included vaccinations, vitamin and mineral supplementation, antioxidant supplementation, or any treatment up to 1 month prior to participation in the study.

Dogs that did not have cardiac murmurs were included in the control group. Dogs that were found to have a grade 2 or 3/6 left or left and right systolic murmur underwent cardiac evaluation. Cardiovascular disease was confirmed by history, clinical examination, standard 9-lead electrocardiogram, and echocardiography with two-dimensional, M-mode, color, and spectral Doppler modes performed by an experienced echocardiographer (ADP; VIVID E9, General Electric Healthcare, Milwaukee, Wisconsin, United States). Myxomatous mitral valve disease was confirmed based on typical valve thickening and mitral regurgitation with normal systolic function, and B1 stage was confirmed when no left ventricular end-diastolic, end-systolic, and left atrial enlargement were noted (1, 33). The dogs never had clinical signs of heart failure. Left ventricular size was measured in M-mode from the right parasternal short axis view at the level of the tip of papillary muscles and left atrium was measured from 2-dimensional right parasternal longitudinal and right parasternal short axis views in early diastole. Left atrial to aortic ratio < 1.6 was considered normal (1).

Venous blood (12 mL) was collected as part of the pre-anesthesia health assessment (6 mL) and to sample for selected oxidative stress parameters (6 mL).

### 2.2. Sampling and sample storage

Tubes containing anticoagulant lithium heparin (2 × 2 mL; Vacuette, Greiner Bio-One, Austria) were used to collect venous blood samples for determination of plasma vitamin E, ACL, and ACW concentrations, the activity of GPX in whole blood, and the activity of SOD in erythrocyte lysate. Tubes containing the anticoagulant EDTA (2 mL; Vacuette, Greiner Bio-One, Austria) were used to collect the blood samples for determination of MDA concentration in plasma. Serum separation tubes (4 mL; Vacuette; Greiner Bio-One, Austria) were used to collect blood samples for determination of serum cholesterol and triglyceride concentrations. Serum separation tubes were kept at room temperature for 30 min before centrifugation at 1,300 × g for 10 min.

Blood samples for the determination of vitamin E, ACW, ACL, and MDA concentrations were centrifuged immediately after collection at 1,500 × g for 15 min at 4°C. Plasma was harvested and frozen at −80°C until batch analyses. For determination of GPX activity, heparinized whole blood was aliquoted into cryovials and immediately frozen at −80°C until batch analysis. Erythrocyte lysates for the determination of SOD activity were prepared immediately after blood collection following the manufacturer’s instructions and stored at −80°C until batch analysis. An automated biochemical analyzer (RX-Daytona, Randox, United Kingdom) was used to measure hemoglobin concentration in the erythrocyte lysate spectrophotometrically by the cyanmethemoglobin method and to measure serum cholesterol and triglycerides concentrations.

### 2.3. Determination of vitamin E concentration in plasma

Plasma vitamin E concentrations were measured by high-performance liquid chromatography (HPLC) with fluorescence detection using an external standard of alpha-tocopherol (Sigma, United States) according to analytical methods described previously (34, 35).

Lipid-standardized vitamin E values were calculated as the ratio between the plasma vitamin E concentration (μmol/L) and the sum of the concentrations of total cholesterol (mmol/L) and triglycerides (mmol/L) in serum (36–38).

### 2.4. Determination of antioxidant capacity of water- and lipid-soluble antioxidants

Plasma ACL and ACW concentrations were measured as previously described (39) using a PHOTOCHEM analyzer and the reagent kits provided (Analytik Jena, Jena, Germany). Results are expressed in nmol equivalents of ascorbic acid in mL of sample for ACW and in nmol equivalents of Trolox (6-hydroxy-2,5,7,8-tetramethylchroman-2-carboxylic acid) in mL of sample for ACL.

### 2.5. Determination of whole blood GPX and erythrocyte SOD activities

The GPX and SOD activities were measured spectrophotometrically using an automated biochemical analyzer (RX-Daytona, Randox, United Kingdom) and commercial Ransel and Ransod kits (Randox, United Kingdom), respectively. We expressed the activities of GPX and SOD as units per gram of hemoglobin (U/g HGB).

### 2.6. Determination of MDA concentration in plasma

Plasma samples for total MDA were derivatized with 2, 4-dinitrophenylhydrazine and assayed using liquid chromatography coupled with tandem mass spectrometry, as described elsewhere (40). The MDA derivative was analyzed using an Agilent 1290 Infinity HPLC coupled to an Agilent 6460 Triple-Quadrupole mass spectrometer equipped with a Jet Stream electrospray ionization source (Agilent Technologies, United States).

### 2.7. Statistical analysis

Commercially available software (IBM SPSS 25.0, Chicago, Illinois, United States) was used for data analysis. The Shapiro–Wilk test was used for assessment of data distribution. For comparison of age, weight, cholesterol, triglycerides, MDA, and antioxidant parameters between groups, a parametric independent samples *t*-test (normally distributed data) and a nonparametric Mann–Whitney test (nonnormally distributed data) were applied. Furthermore, we performed one-way analysis of covariance (one-way ANCOVA) with age and weight as covariates. Correlations between MDA and antioxidant parameters were performed using nonparametric Spearman rank correlation in the case of nonnormally distributed data or parametric Pearson correlation coefficient analysis in the case of normally distributed data. We expressed normally distributed data as means ± standard deviations (SD) and nonnormally distributed data as median and interquartile ranges (IQR-25th to 75th percentiles). A value of *p* < 0.05 was considered significant.

## 3. Results

Twenty-six dogs were included in the study. The MMVD group (*N* = 14) was represented by the following breeds: mixed breed (*N* = 4), Cavalier King Charles Spaniel (*N* = 2), Border Collie (*N* = 1), Beagle (*N* = 1), Bull Terrier (*N* = 1), Pug (*N* = 1), Tibetan Terrier (*N* = 1), Miniature Dachshund (*N* = 1), Irish Setter (*N* = 1), and Flat Coated Retriever (*N* = 1). The control group was represented by the following breeds: mixed breed (*N* = 3), Bull Terrier (*N* = 2), Border Collie (*N* = 1), Labrador Retriever (*N* = 1), Greyhound (*N* = 1), King Charles Spaniel (*N* = 1), Malinois (*N* = 1), German Shepherd (*N* = 1), and Romagna Water Dog (*N* = 1). We found no significant differences in sex and weight between the MMVD and control group. There were 4 intact and 2 neutered female dogs and 6 intact and 2 neutered male dogs in the MMVD group. In the control group, there were 6 neutered female dogs, 4 intact male dogs, and 2 neutered male dogs. The median weight of the dogs was 13.4 kg in the MMVD group (IQR: 10.1–25.7 kg) and 24 kg in the control group (IQR: 16.8–28.4 kg). There was a significant age difference between the MMVD (mean ± SD: 108 ± 38 months) and the control group (mean ± SD: 71 ± 29 months), with the control dogs being significantly younger (*p* = 0.01). One-way ANCOVA showed no effect of age and weight on any of our results.

Antioxidant parameters and MDA, cholesterol, and triglyceride concentrations, including reference values if available, are shown in Table 1. Dogs with MMVD had significantly higher vitamin E, ACL, MDA, and cholesterol concentrations and significantly higher LS-VitE values than control dogs. We found no significant difference between the MMVD and control group with respect to SOD and GPX activities, and ACW and triglyceride concentrations.

**Table 1:** Antioxidant parameters and malondialdehyde, cholesterol, and triglyceride concentrations in the MMVD and control group, and reference values available for each parameter.

	MMVD	Control	*p* ^a^	*p* ^b^	Reference values
Vitamin E (μmol/L; mean ± SD)	70.3 ± 19.6	40.3 ± 11.6	**<0.001**	**<0.001**	56.8; 38.9–72.4^a^, (26)
LS-VitE (x10-3; mean ± SD)	9.17 ± 2.16	6.64 ± 1.84	**0.004**	**0.023**	8.38; 6.77–10.59^a^, (26)
ACL (nmol/mL; mean ± SD)	129.4 ± 28.8	87.6 ± 12.2	**<0.001**	**<0.001**	N/A
ACW (nmol/mL; mean ± SD)	30.3 ± 10.3	30.1 ± 9.3	0.948	0.951	N/A
SOD (U/g HGB; mean ± SD)	2245.8 ± 291.0	2422.5 ± 337.5	0.170	0.112	1409.3–2347.3^b^, (41)
GPX [U/g HGB; IQR (25th–75th percentile)]	455.2 (409.6–478.5)	483.9 (433.3–529.5)	0.114	0.412	349.4–705.3^b^, (41)
MDA (μmol/L; mean ± SD)	5.47 ± 1.00	4.44 ± 0.54	**0.004**	**0.036**	N/A
Cholesterol (mmol/L; mean ± SD)	6.87 ± 1.23	5.42 ± 1.37	**0.009**	**0.004**	3.50–6.99^c^
Triglycerides [mmol/L; IQR (25th–75th percentile)]	0.83 (0.63–1.03)	0.57 (0.46–0.85)	0.064	0.694	0.2–1.3^c^

*p*-values less than 0.05 were considered significant (bold). *p*^a^, independent samples *t*-test or Mann–Whitney test. *p*^b^, one-way ANCOVA with age and weight as covariates. SD, standard deviation; LS-VitE, lipid-standardized vitamin E calculated as the ratio between vitamin E concentration and the sum of cholesterol and triglyceride concentrations; ACL, antioxidant capacity of lipid-soluble antioxidants; ACW, antioxidant capacity of water-soluble antioxidants; SOD, superoxide dismutase; GPX, glutathione peroxidase; MDA, malondialdehyde; MMVD, myxomatous mitral valve degeneration; IQR, interquartile range; HGB, hemoglobin concentration; ^a^Median; minimum to maximum values; ^b^percentile range (5%–95%); ^c^reference range, according to the diagnostic laboratory at the Small Animal Clinic of the Veterinary Faculty of Ljubljana; N/A, not available.

Correlation analysis showed no significant correlations between MDA and antioxidant status parameters in either group of dogs.

## 4. Discussion

The results of the present study suggest that antioxidant status is altered but not depleted and that oxidative damage to lipids is already present in dogs with MMVD ACVIM B1. According to our results, the antioxidant response to increased oxidative damage consisted of the mobilization of lipid-soluble antioxidants, while water-soluble antioxidants and antioxidant enzymes remained unchanged.

The dogs in the MMVD group were significantly older compared with those in the control group. It is difficult to determine the extent to which the age difference between control and MMVD dogs may have influenced the results of our study; however, the results of one-way ANCOVA with age and weight as covariates showed no influence of these two covariates on our results. The significant age difference between dogs with cardiovascular disease, including MMVD, and healthy dogs has been noted in similar studies (8, 9, 25, 26, 28, 31). The disease is usually diagnosed in middle-aged to older dogs (2) but may also affect young adult dogs of predisposed breeds (e.g., Cavalier King Charles Spaniel) (1, 3), which were represented by only a few dogs in this study. The influence of age is explained in more detail in the following sections for each of the measured parameters.

The concentration of vitamin E and the level of LS-VitE were significantly higher in the MMVD group compared with the control group; their levels were in general agreement with previously published data in healthy dogs and dogs with cardiovascular disease (26) (Table 1). Significantly higher vitamin E and LS-VitE levels in MMVD dogs could be due to the mobilization of vitamin E from storage organs as part of the compensatory response to the increased production of ROS in dogs with MMVD (17, 42). Lipid standardization of plasma vitamin E concentration excludes the influence of triglycerides and cholesterol (36–38), the latter being significantly higher in dogs with MMVD, as previously reported (25). Contrary to our findings, another study found no significant difference in the concentrations of vitamin E and cholesterol in dogs with early-stage valvular heart disease compared with control dogs (43). However, in that study, feeding a diet enriched with antioxidants, including vitamin E, significantly improved the echocardiographic parameters in dogs with chronic valvular heart disease (43). Likewise, in a recent study, a significant reduction in left atrial size and mitral regurgitation was observed in dogs with MMVD ACVIM B1 and B2 that were fed a cardioprotective mixture containing vitamin E (44).

Contrary to our findings, no significant differences in vitamin E concentration and levels of LS-VitE were observed in dogs with different stages of heart disease and heart failure compared with healthy dogs (26), or in King Charles Spaniels with different stages of MMVD (25). These results are difficult to compare with the present study because of differences in study design and type of classification of cardiac disease (ISACHC, International Small Animal Cardiac Health Council) used by other authors (26).

Studies in humans showed a linear increase in plasma vitamin E concentration with age (45, 46). In dogs, however, a study of pair-fed 5–10-year-old Labrador dogs showed a decrease in serum vitamin E concentration by 8 years of age that could be as high as 50%. No vitamin E deficiency was present, and the authors concluded that this decline may be part of the aging process (47). This is in contrast to our results, in which dogs with early-stage MMVD that were significantly older than the control group had significantly higher vitamin E concentrations.

In our study, ACL levels were significantly higher in the MMVD group compared with the control group. These results cannot be compared with reference ranges because they have not yet been reported in dogs. The ACL consists of exogenous and endogenous lipophilic antioxidants, including vitamin E, coenzyme Q_10_, and carotenoids (48, 49). Vitamin E accounts for 75% of lipid-soluble antioxidants in human plasma (48) and, together with other lipid-soluble antioxidants, may have also contributed to the significantly higher ACL levels in our MMVD group. Recent studies have confirmed the involvement of lipid soluble antioxidants, especially coenzyme Q_10_, in dogs with MMVD (30, 31). Significantly higher baseline concentrations of coenzyme Q_10_ were found in dogs with MMVD ACVIM B2 compared with healthy controls (31), while reduced myocardial coenzyme Q_10_ concentrations were found in Cavalier King Charles spaniels with heart failure due to MMVD (50). Coenzyme Q_10_ is an endogenous lipid-soluble antioxidant involved in mitochondrial energy metabolism and, like vitamin E, is a potent free radical scavenger that prevents lipid peroxidation of cell membranes (51). Considering the key role of coenzyme Q_10_ in cellular energy production, and the high energy requirements of cardiac cells, coenzyme Q_10_ has a potential role in the prevention and treatment of heart ailments by improving cardiac bioenergetics (50, 52, 53).

No significant differences in SOD and GPX activity were found between the two groups studied. This is in general agreement with our previously published studies investigating the activities of SOD and GPX in dogs with cardiovascular diseases (28) and healthy dogs (41). In the present study, the activities of SOD and GPX in the MMVD and control group were consistent with our previously published data in healthy dogs (Table 1) (41).

Similarly, erythrocyte SOD did not differ significantly in dogs with idiopathic dilated cardiomyopathy (IDCM) compared with healthy dogs in another study (54). Other authors evaluated serum extracellular SOD-3, catalase, and glutathione reductase activities and total antioxidant capacity (TAC) in healthy dogs and those with different stages of MMVD (27). Serum SOD activity was significantly lower only in dogs with MMVD ACVIM stage C compared with dogs with ACVIM B and healthy dogs, while glutathione reductase activity remained unchanged. On the other hand, catalase activity was significantly higher in dogs with MMVD ACVIM B compared with healthy dogs and those with MMVD ACVIM C (27). These results are difficult to compare with ours because of differences in the types of samples and the analytical methods used.

Glutathione peroxidase activity did not differ between dogs with heart disease and healthy dogs in some other studies (26, 31), while it was significantly higher in dogs with MMVD and dilated cardiomyopathy (DCM) without congestive heart failure compared with healthy dogs (9). Significantly increased erythrocyte GPX activity was also reported in dogs with IDCM compared with healthy dogs (54). The pathophysiology of MMVD differs from that of other cardiac diseases such as DCM. Although oxidative stress and inflammation have been shown to be involved in heart disease (8, 9), the antioxidant response may differ depending on the type of disease (27). According to the results of the present study, the activities of antioxidant enzymes SOD and GPX were not triggered by the increased production of ROS in dogs with early-stage MMVD.

The correlation between levels of antioxidant enzymes (SOD, GPX) and oxidative damage during aging has been demonstrated in humans (55–57). There are few reports on the effects of age and/or sex on antioxidant enzyme activity in dogs, and the reports are conflicting. One study found a significant positive effect of age on the activity of erythrocyte SOD, but no effect on the activity of whole blood GPX and plasma TAC, and no effect of sex on TAC, SOD, and GPX (41). Other studies have found significantly higher activity of SOD in erythrocyte lysate (58) and plasma (59) in old dogs compared with younger dogs. Significantly higher erythrocyte GPX activity was found in old male and female dogs compared to young male and female dogs (58). In the same study, a significant difference in GPX activity was found between old male and female dogs, whereas there was no significant difference between young male and female dogs (58). A significant increase in whole blood GPX activity (not standardized to hemoglobin concentration) with age was found in Labrador retrievers (47). On the other hand, another study found no significant difference in plasma GPX activity between old (8–13 years) and younger dogs (0–7 years) (59).

In our study, plasma MDA concentrations were significantly higher in the MMVD group compared with the control group, indicating an increased degree of lipid peroxidation due to increased oxidative stress in dogs with MMVD. Malondialdehyde is one of the most used markers of lipid peroxidation and thus oxidative stress (14, 15, 60, 61). However, the methods used to determine its concentration in biological samples vary widely, leading to incomparable results between studies. Increased MDA concentrations in cardiac tissue have been reported in dogs with volume overload-mediated heart failure due to experimentally induced cardiac regurgitation (62). Other studies reported no significant differences in MDA levels between healthy dogs and dogs with MMVD and DCM without congestive heart failure (9, 26), between healthy dogs and dogs with IDCM (54) and between healthy dogs and dogs with heart failure due to DCM or chronic valvular disease (23). However, in the latter study, a significantly higher concentration of 8-F2alpha-isoprostanes, another marker of lipid peroxidation, was found in dogs with heart failure compared with healthy dogs (23).

Lipid peroxidation has been studied as a factor in the human aging process (63). To clarify the influence of aging on lipid metabolism, a study was conducted in dogs in which plasma MDA was measured along with other parameters (59). The study was conducted on two groups of dogs, 0–7 years and 8–13 years of age. The results of the study showed no significant difference in MDA concentration between old and younger dogs; however, the authors reported a moderate positive significant correlation between MDA and the age of the dogs (59). In a study of oxidative damage in a canine model of human brain aging, an age-related increase in serum MDA was found to correlate with the accumulation of MDA in the prefrontal cortex of the brain (64). In the present study, the dogs in the MMVD group, which had significantly higher plasma MDA concentrations than the control dogs, were also significantly older than the control dogs. However, a one-way ANCOVA with age as well as weight as covariates revealed no effect of these two covariates on the statistically significant difference in MDA concentration between the two groups.

Contrary to our expectations, we found no significant correlations between the degree of lipid peroxidation, as measured by MDA concentration, and parameters of antioxidant status in both groups. Similarly, in a previous study, no correlation was found in the untreated dogs with MMVD and DCM, whereas a positive correlation was found in dogs receiving treatment (26). Other studies have found no correlation between plasma MDA concentration and the clinical stage of MMVD (23, 25). However, when MDA was placed in the context of the inflammatory response in dogs with heart disease, a significant positive correlation between N-terminal pro-B type natriuretic peptide and MDA concentrations was found in dogs with heart failure (9).

According to the results of the present study, we can assume that despite the significantly higher MDA concentration found in our MMVD group compared with the control group, MDA alone may not be the best parameter for the evaluation of oxidative lipid damage (23, 25). In future studies, assessment of lipid peroxidation should include determination of both MDA and 8-2alpha isoprostane concentrations.

In our study, there was no significant difference in ACW between the two groups, while ACL was significantly higher in the MMVD group. Total antioxidant capacity estimates the overall antioxidant status of a subject and was found to be significantly lower in dogs with heart failure compared to healthy controls (8). Although TAC may provide insight into individual overall antioxidant status (8, 9), assessment of ACL and ACW may be a better method to clarify which antioxidant compartment was activated under specific circumstances (65). According to our results, the homeostasis of water-soluble antioxidants represented by hydrophilic antioxidants such as vitamin C, uric acid, glutathione, proteins, and other low molecular weight antioxidants (66, 67) was not altered in dogs with early-stage MMVD.

The authors acknowledge certain limitations of the study. First, dogs in the control group were significantly younger than those in the MMVD group. We wanted to include age-matched control dogs because of the possible effects of age on antioxidant enzyme activity (41, 47, 58) and vitamin E (47), but this was not possible because of various health conditions in older dogs that precluded their inclusion in the control group. We performed the one-way ANCOVA with age and weight as covariates. Statistical analysis showed no significant effect of age and weight on our results. Second, the effect of diet on measured oxidative status parameters cannot be excluded, although it has been reported that no detectable differences in oxidative stress parameters such as vitamins E and C, antioxidant capacity, retinol, MDA, 8-F2alpha isoprostane, protein carbonyls, reduced and oxidized glutathione were found between dogs fed antioxidant-enriched diets compared with other diets (23). According to the owners’ responses, the dogs in our study were fed a random diet (homemade or commercial food or a combination of both) with composition that met standard nutritional requirements; however, the information provided in the questionnaire may not correspond to the actual dietary intake.

## 5. Conclusion

The results of this study suggest that antioxidant status is altered in dogs with MMVD stage B1 and consists mainly of the activation of fat-soluble antioxidants. Water-soluble antioxidant capacity and the activity of intracellular antioxidant enzymes SOD and GPX remain unchanged in dogs with early-stage MMVD. In addition, significantly higher MDA levels in dogs with MMVD indicate the presence of oxidative damage to lipids and thus increased oxidative stress. Further studies in a larger number of age and sex-matched dogs are needed to evaluate selected antioxidant and lipid peroxidation parameters in dogs with early-stage MMVD.

## Data availability statement

The raw data supporting the conclusions of this article will be made available by the authors, without undue reservation.

## Ethics statement

All procedures involving the use of animals were approved by the National Ethics Committee of the Ministry of Agriculture, Forestry and Food, Veterinary Administration of the Republic of Slovenia (license No U34401-38/2013/2, approval date 30.7.2013). All procedures were conducted in accordance with the Slovenian Animal Protection Act (The Official Gazette of the Republic of Slovenia, 43/2007). The studies were conducted in accordance with the local legislation and institutional requirements. Written informed consent was obtained from the owners for the participation of their animals in this study.

## Author contributions

ANS, AN, AS, and KT contributed to the conception and design of the study. ADP, AN, and KT performed recruitment and clinical examinations of the dogs. ANS, TP, TV, and VR performed laboratory analyzes. ANS and KT organized the database. ANS performed the statistical analysis. KT wrote the first draft of the manuscript. AN, ANS, TP, TV, and VR wrote sections of the manuscript. AS was responsible for project oversight, administration, and fund acquisition. All authors contributed to the article and approved the submitted version.

## Funding

This research, including APC, was funded by the Slovenian Research Agency (ARRS), grant nos. P4–0053, P4–0097, and P1–0189.

## Conflict of interest

The authors declare that the research was conducted in the absence of any commercial or financial relationships that could be construed as a potential conflict of interest.

## Publisher’s note

All claims expressed in this article are solely those of the authors and do not necessarily represent those of their affiliated organizations, or those of the publisher, the editors and the reviewers. Any product that may be evaluated in this article, or claim that may be made by its manufacturer, is not guaranteed or endorsed by the publisher.
